# Improving the Wet-Spinning and Drawing Processes of Poly(lactide)/Poly(ethylene furanoate) and Polylactide/Poly(dodecamethylene furanoate) Fiber Blends

**DOI:** 10.3390/polym14142910

**Published:** 2022-07-17

**Authors:** Claudia Fabris, Davide Perin, Giulia Fredi, Daniele Rigotti, Mauro Bortolotti, Alessandro Pegoretti, Eleftheria Xanthopoulou, Dimitrios N. Bikiaris, Andrea Dorigato

**Affiliations:** 1 Department of Industrial Engineering and INSTM Research Unit, University of Trento, Via Sommarive 9, 38123 Trento, Italy; claudia.fabris95@gmail.com (C.F.); daniele.rigotti-1@unitn.it (D.R.); mauro.bortolotti@unitn.it (M.B.); alessandro.pegoretti@unitn.it (A.P.); andrea.dorigato@unitn.it (A.D.); 2 Laboratory of Polymer Chemistry and Technology, Department of Chemistry, Aristotle University of Thessaloniki, GR-541 24 Thessaloniki, Greece; elefthxanthopoulou@gmail.com (E.X.); dbic@chem.auth.gr (D.N.B.)

**Keywords:** poly(lactic acid), poly(ethylene 2,5 furanoate), poly(dodecamethylene 2,5−furandicarboxylate), blends, fibers, mechanical properties

## Abstract

This work aims to produce poly(lactic acid) (PLA)/poly(alkylene furanoate)s (PAF)s fiber blends for textile applications and evaluates their microstructural, chemical, thermal, and mechanical properties. The work focuses on two PAFs with very different alkyl chain lengths, i.e., poly(ethylene 2,5−furandicarboxylate) (PEF) and poly(dodecamethylene 2,5−furandicarboxylate) (PDoF), which were blended in solution at various concentrations (in the range 2.5–10 wt %) with PLA, wet spun, and subsequently drawn. Light optical micrographs highlight that PLA/PEF blends present large and concentrate PEF domains, whereas PLA/PDoF blends show small and homogeneously distributed PDoF domains. The blends appear to be immiscible, which is confirmed also by scanning electron microscopy (SEM), Fourier−Transform Infrared (FT−IR) spectroscopy, and differential scanning calorimetry (DSC). Thermogravimetric analysis (TGA) highlights that the addition of the PAFs improves the thermal stability of the fibers. The drawing process, which was carried out at 80 °C with a heat setting step at 95 °C and at three draw ratios, improves the mechanical properties of the fibers upon the addition of the PAFs. The results obtained in this study are promising and may serve as a basis for future investigations on these novel bio−based fiber blends, which can contribute to increase the environmental sustainability of industrial textiles.

## 1. Introduction

Since the synthesis of the first polymer matrices, plastics have seen a pervasive increase in use from the 19th century to the present day [1,2,3]. The first concerns regarding their consumption were raised in the 1960s, when plastic debris was found in the ocean for the first time. Nonetheless, over the last 50 years, synthetic polymer production has increased by 20 times with approximately 5 billion tons produced up to 2015 [4,5]. This has led to a surge in the demand for energy and resources with approx. 8% of the total fossil fuel oils utilized to manufacture polymers [6]. Hence, although plastics are incredibly versatile materials and are often the greenest choice for several applications, it is undoubted that the production and disposal of petroleum−based plastics are linked to global warming, pollution, waste accumulation, and fossil fuel depletion [7,8,9].

To help alleviate the dependence on fossil fuels and encourage a more sustainable development, a possible way is represented by biopolymers, which are bio−derived and/or biodegradable [10,11]. Among the various biopolymers; one of the most well−known is poly(lactic acid), or PLA, an aliphatic polyester derived from the direct condensation of lactic acid or by the ring−opening polymerization of lactide, which can be both derived from the fermentation of dextrose from renewable biomass [12]. PLA is an interesting alternative to conventional commodity polymers for textile and packaging applications, thanks to its high tensile modulus (approx. 3 GPa), high mechanical strength (approx. 40 MPa), recyclability, and compostability [13,14,15,16,17]. On the other hand, PLA shows a low heat deflection temperature, it is prone to hydrolysis in the presence of moisture, it is characterized by relatively high brittleness, and it is highly hygroscopic, resulting in a premature loss of mechanical properties.

To mitigate these drawbacks, a largely employed technique is polymer blending, which consists of physically mixing two or more polymers to obtain improved and synergistic properties compared to those of the starting materials. This is a more economically feasible solution to tailor the final required properties compared to synthesizing a new polymer. Depending on the miscibility of the constituents, the blends can result as immiscible, compatible, or miscible, thereby leading to different morphologies and different physical properties [18,19].

Some recent studies have shown the potential of relatively new classes of polymers, such as poly(alkylene furanoate)s (PAFs), to be blended with PLA. PAFs are mainly produced by the polycondensation of furandicarboxylic acid (FDCA), which is obtained via the acid−catalyzed dehydration of polysaccharides [20,21] and different diols [22,23,24]. Among the FDCA−based polyester family, poly(ethylene 2,5−furandicarboxylate) (PEF) is the most famous. PEF is referred to as the furan counterpart of poly(ethylene terephthalate) (PET) [25] and can hence be regarded as the renewable equivalent of PET. It is produced by the polycondensation of FDCA with ethylene glycol and presents an elastic modulus of 2–3 GPa, a tensile strength of 40–70 MPa, and superior barrier properties to PET, as PEF is 10 times less permeable to oxygen and 20 times less permeable to carbon dioxide than PET [26]. On the other hand, due to its brittleness and especially the still prohibitive production costs, industrial production is still in its infancy. PEF is not the only furanoate polyester of industrial and commercial interest. Furan−based polymers with longer−chain diols have also been produced by using subunits with a linear carbon chain spanning from C_3_ to C_12_, which has led to the synthesis of poly(1,3−propylene 2,5−furandicarboxylate) (PPF), poly(1,4−butylene 2,5−furandicarboxylate) (PBF), poly(1,6−hexylene 2,5−furandicarboxylate) (PHF) and poly(1,8−octylene 2,5−furandicarboxylate) (POF) by Jiang et al. [27] and of poly(pentylene 2,5−furandicarboxylate) (PPeF), poly(nonylene 2,5−furandicarboxylate) (PNF), poly(decylene 2,5−furandicarboxylate) (PDeF), and poly(dodecylene furandicarboxylate) (PDoF) by Bikiaris et. al. [28,29]. The number of methylene groups in the aliphatic chain greatly influences the microstructural and thermomechanical properties of the furanoates [28]: as the number of methylene groups increases, the molecular mobility also increases, thereby decreasing the stiffness, strength, glass transition temperature, and melting point [30]. 

The production of PLA/furanoate polyesters fiber blends for textile applications could help mitigate the shortcomings of PLA and improve the sustainability of textile products by developing a valid alternative to current fossil−fuel−based synthetic fibers and textiles. In fact, PLA has been already used in the production of sportswear, furnishings, drapes, and non−wovens, although further improvements in the moisture resistance and mechanical properties are required [31]. In previous works of our group, PAFs have already been blended with PLA to produce wet−spun fibers, which showed promising improvements in the mechanical properties, thermal stability, and water absorption tendency [22,24,32]. Those works demonstrated that PLA/PAF blends with a small fraction of PAFs (max. 10−20 wt %) showed the best combination in terms of good thermomechanical properties and limited water absorption tendency, especially after drawing. However, in those works, the irregular morphology and high porosity of the produced fibers, derived from the non−optimized processing equipment and parameters, resulted in considerable data scattering. Moreover, those works failed in highlighting how the physical−mechanical properties of the resulting fibers were affected by the alkyl chain length.

Hence, this study focuses on the optimization of the production of PLA/PEF and PLA/PDoF blends for textile purposes via wet spinning and subsequent drawing. The two selected PAFs, i.e., PEF and PDoF, have an alkyl chain subunit containing 2 and 12 carbon atoms, respectively, thus being the PAFs with the shortest and the longest alkyl chain available. The blend with PEF will eventually produce a fiber possessing high mechanical properties coupled with high barrier properties. On the other hand, blending PLA with PDoF will produce a fiber reporting higher toughness in comparison to neat PLA and remarkable barrier properties. The blends were developed by preparing PLA/PAF solutions with a PAF content in the range 2.5–10 wt %, which were wet spun with an improved version of the previously used lab−made spinning device and then drawn at different draw ratios. The prepared fibers were subsequently characterized to evaluate the microstructural, chemical, thermal, and mechanical properties as a function of the PAF type and concentration as well as the applied draw ratio.

## 2. Materials and Methods

### 2.1. Materials

Ingeo^®^ 4032D PLA (density = 1.24 g/cm^3^, MFI at 210 °C and 2.16 kg = 7 g/10min) was supplied in pellet form by NatureWorks LLC (Minnetonka, MN, USA). Two different poly(alkylene furanoate)s were employed to produce fiber blends. The first is poly(ethylene furanoate) (PEF), which is a fully bio−based polymer with an intrinsic viscosity value of 0.45 dL/g, a T_g_ of 75–80 °C, a T_m_ at 210–215 °C and a degradation temperature at approx. 400 °C [33,34]. The second is poly(dodecylene furanoate), which was synthesized at a lab−scale by a two−stage melt polycondensation method using 1,12−dodecanediol and 2,5−dimethylfuran−dicarboxylate (DMDF). This polymer presents an intrinsic viscosity of 0.49 dL/g a T_g_ of −5 °C and a T_m_ of 111 °C [35]. Chloroform, 1,1,1,3,3,3−hexafluoro−2−propanol (HFIP) and methanol (purity = 99.9%) were purchased from Carlo Erba Reagents s.r.l (Milan, Italy). Both PEF and PDoF were provided in the form of thin flakes and were used as received.

### 2.2. Fiber Spinning

**Figure 1:**
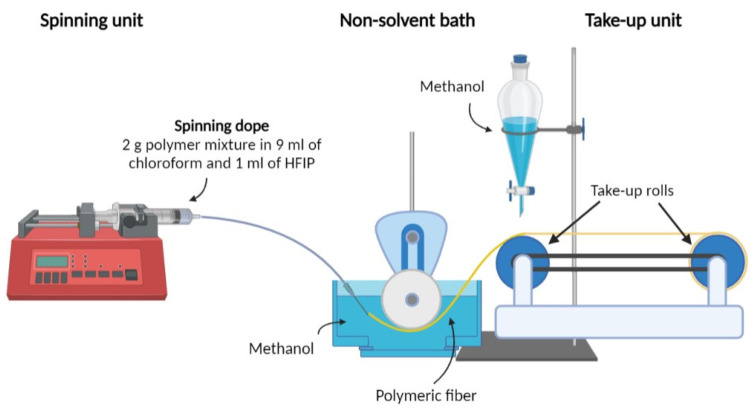
Schematization of the wet spinning set−up used for fiber production.

Fiber blends were produced through wet spinning. Neat PLA, PLA/PEF, and PLA/PDoF mixtures were dissolved in a mixture of chloroform and HFIP. The ability of chloroform to dissolve both PLA and furan−based polyesters with long alkyl chains (e.g., PDoF) was previously reported [22,24,32,34], while HFIP was necessary to provide the dissolution of PEF [36]. The wet−spinning process was used to produce neat PLA fibers, PLA/PEF, and PLA/PDoF fiber blends with PEF and PDoF concentrations equal to 2.5 wt %, 5 wt %, and 10 wt %. The samples were labeled as PLA, PEF−x, or PDoF−x, where x represents the weight fraction of PEF or PDoF and is equal to 2.5%, 5%, or 10%. The spinning dope was produced by dissolving 2 g of polymer mixtures in 9 mL of chloroform and 1 mL of HFIP, which resulted in the perfect mixtures for dissolving PLA/PEF blends, as reported in our previous work [36]. The mixture was magnetically stirred for 3 h at 50 °C and subsequently sonicated for 10 min in a Labsonic LBS1 bath (Falc Instruments S.r.l, Treviglio, Italy) to remove any air bubbles before being poured into a glass syringe. The wet−spinning process was carried out with the lab−made device shown in Figure 1. The glass syringe was connected to a stainless steel, 18−gauge needle through a polytetrafluoroethylene (PTFE) tube and mounted on a Harvard Apparatus 11 plus syringe pump (Harvard apparatus Inc., Holliston, MA, USA). The spinning dope entered a non−solvent bath to remove the solvents. The flow of the coagulated dope was guided by a PTFE wheel in the methanol bath and then transferred to the collection system composed of an intermediate and collection of (PVC) rolls with a diameter of 5 cm. A separatory funnel placed methanol drops on the intermediate roll to avoid the fiber sticking to the latter. The non−solvent bath was composed of methanol, which was observed to induce PLA crystallization [22,24,37]. 

Following some preparatory trials, the spinning rate was set to 0.05 mL/min, whereas the rotating speeds of the PTFE wheel and the collection system rolls, which were controlled through an Arduino Mega board, were 3 rpm and 12 rpm, respectively. The above−described process parameters allowed a correct solvent diffusion avoiding thus fiber rupture, and they imparted a spinning draw ratio (SDR) of 8, which was calculated as reported in Equation (1):

(1) $$SDR=\frac{v_{feeding}}{v_{collection}}$$

where $v_{feeding}$ and $v_{collection}$ are the spinning dope feeding and fiber collection velocities, respectively.

### 2.3. Fiber Drawing

**Figure 6:**
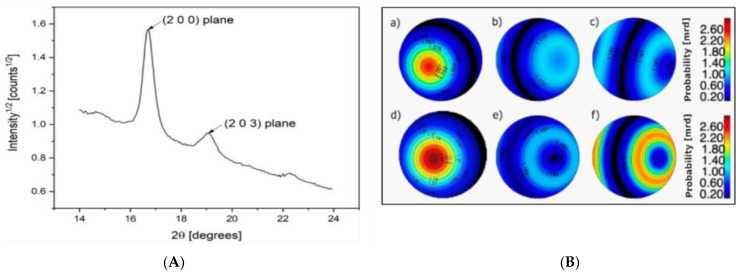
(**A**) Example of a 1D XRD diffraction pattern of a PLA−as sample, (**B**) polar figures showing the preferred orientations of the (200) plane for (**a**) PLA−as, (**b**) PEF−10%−as, (**c**) PDoF−10%−as, (**d**) PLA−DR2, (**e**) PEF−10%−DR2, and (**f**) PDoF−10%−DR2 fibers. Equal area projection on the fiber axis plane; pole density scale expressed in M.R.D.

Drawing was carried out to further improve the mechanical properties of the prepared samples. Ten fibers per composition were glued on a paper frame with a gauge length of L_0,D_ = 50 mm, which was subsequently mounted on an Instron^®^ 5969 electromechanical testing machine equipped with an Instron^®^ 3119−409 environmental chamber and a 100 N load cell. The temperatures selected for the drawing process were chosen according to the DSC analysis performed on the constituents of the blends (see Figure 6). Thus, these temperatures enable improving the deformability of the fibers, also avoiding the melting of the PDoF constituent. The drawing procedure was carried out by following the optimized parameters reported in previous work [32], i.e., by keeping the samples at 80 °C for 10 min to stabilize the temperature and then drawing them at three different draw ratios (1, 2, and 4), corresponding to once, twice, and four times the initial fiber length, at a speed of 50 mm/min. The fibers with a draw ratio of 1 were kept at 80 °C for 15 min, allowing all prepared specimens to be subjected to the same thermal treatment. The samples were subsequently heat−set at 95 °C for 2 min to prevent any further deformation of the specimen upon removal from the testing apparatus, following the indications reported in previous works on fiber drawing [38]. The complete labels assigned to the samples are summarized in Table 1.

### 2.4. Experimental Techniques

#### 2.4.1. Microstructural Properties

Light optical microscope (LM) micrographs were obtained with a− model Axiophot, Carl Zeiss EL−Einsatz optical microscope (Carl Zeiss AG, Jena, Germany). The LM images of the fiber cross−sections were acquired by winding the fibers around poly(methyl methacrylate) (PMMA) supports, which were embedded in epoxy and subsequently ground with a sequence of abrasive papers (400, 800, 1200, 4000) and polished using 3 µm and 1 µm polishing cloths by following a protocol similar to that reported in our previous work [39]. ImageJ^®^ software (National Institutes of Health campus, Bethesda, MD, USA, release 1.8) was used to measure the fiber diameter and circularity (C), which is a parameter used to determine how close the cross−sections of the fibers were to a perfect circle, and it can be calculated as reported in Equation (2).

(2) $$C=\frac{4\pi A}{p^{2}}$$

where *A* and *p* are the area and perimeter of the fiber, respectively. Additionally, the lateral surface and the cross−sectional cryofracture surface of the fibers were also investigated through a Zeiss Supra40 Field−Emission Scanning Electron Microscope (FESEM) (Carl Zeiss AG, Oberkochen, Germany) after Pt/Pd 80/20 alloy sputtering. 

X−ray diffraction (XRD) measurements were performed on a Huber diffractometer equipped with a microfocus Cu−anode source coupled to a 2D multilayer optics, resulting in a monochromatic, parallel beam and a 1 × 1 mm spot on the sample. A 4 circle goniometer (ω, θ, χ, and φ movements) and a Dectris Eiger 1M 2D hybrid pixel detector (Baden−Dättwil, Switzerland) were also mounted in the set−up. Measurements were performed in transmission mode (fibers orthogonal to the beam) over the 5–60° 2θ range; to determine the preferred orientation, diffraction data were collected over different χ and φ fiber orientations for a total of 36 patterns per sample. Rietveld analysis was carried out using the software Maud^®^ (Version 2.0) [40] implementing the Standard Functions method to model the orientation distribution function of the crystalline fraction [41]. Fourier−transform infrared (FT−IR) spectroscopy was performed in attenuated total reflectance (ATR) mode by a Perkin−Elmer Spectrum One instrument (Perkin Elmer GmbH, Waltham, MA, USA), equipped with a ZnSe crystal operating at a wavenumber interval of 4000–650 cm^−1^ with a resolution of 4 cm^−1^. A total of 100 scans were superimposed per specimen to improve the signal−to−noise ratio.

#### 2.4.2. Thermal Properties

Differential scanning calorimetry (DSC) was carried out using a Mettler^®^ Toledo DSC30 (Mettler Toledo, Columbus, OH, USA) with a TC15 TA controller at 10 °C/min between −50 and 250 °C under a 100 mL/min nitrogen flow. Approx. 3.5 g for each specimen was sealed in aluminum crucibles and subjected to a three−step thermal cycle composed of a first heating scan, a cooling scan, and a second heating scan. The glass transition temperature (T_g_), melting temperature (T_m_), cold crystallization temperatures (T_cc_), and the corresponding specific enthalpy values (Δ*H_m_* and Δ*H_cc_*) of the polymer phases were gathered. The degree of crystallinity of the specimens was calculated as reported in Equation (3):

(3) $$\chi =\frac{\Delta Hmx-\Delta Hccx}{\Delta Hmx^{*}\cdot \omega_{x}\;}\;100\;[\%]$$

where Δ*H_mx_* is the melting enthalpy of the x phase, Δ*H_ccx_* is the enthalpy of cold crystallization of the x phase, ΔH_mx_* is the theoretical enthalpy of melting of the *x* phase, and *ω_x_* is the weight fraction of the x phase, with *x* = PLA, PEF or PDoF. The theoretical enthalpy values of melting ΔH_mx_* was equal to 93.7 J/g for PLA [42], 140 J/g for PEF [30], and 158 J/g for PDoF [35].

Thermogravimetric analysis (TGA) was performed using a Mettler^®^ TG50 thermobalance by testing approximately 7 mg of each specimen at a 10 °C/min rate from 25 to 700 °C in a 100 mL/min nitrogen flow. This test allowed the determination of the mass loss at 150 °C (Δ*m*_150 °C_), which allowed determining the moisture and solvent mass loss, the onset degradation temperature (T_onset_), and the degradation temperatures (T_D_), which are considered at the peak of the derivative thermogravimetry (DTG), and the total mass residue (*m_final_*).

#### 2.4.3. Mechanical Properties

Quasi−static tensile tests were performed by mounting single fibers on a paper frame with a gauge length of 20 mm on an Instron^®^ 5969 electromechanical testing machine (Instron, Nordwood, MA, USA) equipped with a 10 N load cell. Tensile tests were performed at room temperature at a cross−head speed of 2 mm/min and a preload of 0.005 N. This test allowed the determination of the elastic modulus (E), the maximum stress (σ_max_), and the strain at break (ε_b_). Additionally, the tenacity of the produced fibers was calculated as the ratio between the maximum tensile load and the titer of the fibers (see Equation (4)), which in turn was calculated as the ratio between a fixed fiber length (*L_fiber_*) and its mass (*m_fiber_*) (see Equation (5)).

(4) $$Tenacity=\frac{Pmax}{\mathrm{Titer}\;}\;[\mathrm{cN}/\mathrm{tex}]$$

(5) $$Titer=\frac{L_{fiber}}{m_{fiber}\;}\;[g/\mathrm{km}\;\mathrm{or}\;\mathrm{tex}]$$

## 3. Results and Discussion

### 3.1. Microstructural Properties

**Figure 2:**
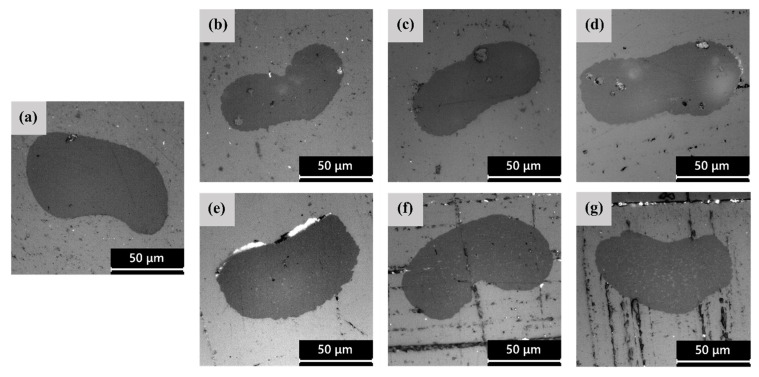
LM micrographs of the polished cross−section of the as−spun fiber blends (**a**) PLA, (**b**) PEF−2.5%−as, (**c**) PEF−5%−as, (**d**) PEF−10%−as, (**e**) PDoF−2.5%−as, (**f**) PDoF−5%−as, (**g**) PDoF−10%−as.

Figure 2a–g show the LM micrographs of the cross−sections of the as−spun fibers, while the average fiber diameters and their circularity C are reported in Table 2. The cross−sections of the fibers present a non−circular shape, which is similar to what has been observed in our previous studies on the wet spinning of PLA/PAF fibers [24,43,44]. This could be the result of the deformation of the fibers, softened by the residual solvent, when collected on the take−up rolls. The crenulation effect observed in some fibers, which is formed as the fibers enter the non−solvent bath due to the kinetics of solvent removal, leads to a larger surface area from which the solvent can be extracted. This effect is more evident for all PLA/PAF fibers than for neat PLA ones, which may suggest an impact of both PEF and PDoF on the solvent removal rate. Moreover, PEF domains in PLA−PEF fibers (Figure 2b–d) are considerably larger than the PDoF domains in PLA−PDoF fibers (Figure 2e–g), which are fine and well−distributed, and their size increases with the distance from the fiber axis, as observed in our previous work [22,24]. The difference between the size of PEF and PDoF domains can be attributed to the different solubility of PEF and PDoF in the solvent mixture. Since PEF is less soluble than PDoF and especially than PLA in the selected solvent mixture, when the spinning dope enters the non−solvent bath, the rate of solvent extraction is probably higher for PLA/PEF mixtures and in particular for the PEF phase, thereby favoring the formation of solvent−poor coalescent PEF domains in a solvent−rich PLA matrix. Conversely, since PLA and PDoF show similar affinity to the chloroform/HFIP solvent mixture and similar solvent extraction rate, this leads to the formation of small and well−distributed PDoF domains. This phenomenon is accentuated where the solvent−extraction rate is slower, i.e., close to the fiber axis, and this also explains the decreasing domain size in this region compared to the cross−section peripheries. 

Lastly, the addition of PAFs leads to a decrease of approx. 46% in the average fiber diameter compared to the PLA fibers and also to an increase in circularity values from approx. 0.80 for the PLA−as sample to >0.90 for all the blends.

**Figure 3:**
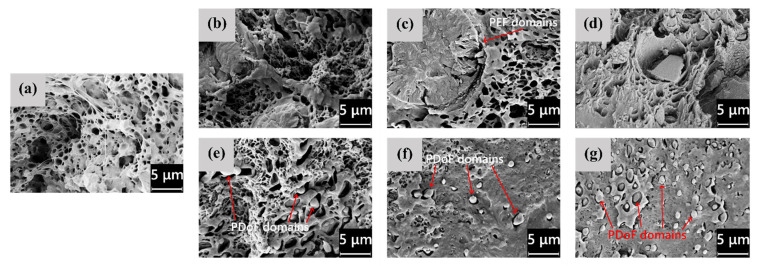
FESEM micrographs of the cross−section of as−spun fibers: (**a**) PLA, (**b**) PEF−2.5%−as, (**c**) PEF−5%−as, (**d**) PEF−10%−as, (**e**) PDoF−2.5%−as, (**f**) PDoF−5%−as, (**g**) PDoF−10%−as.

**Figure 4:**
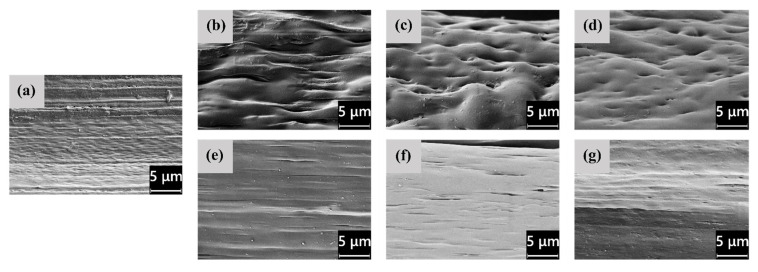
FESEM micrographs of the cryo−fractured cross−section of the as−spun fibers: (**a**) PLA, (**b**) PEF−2.5%−as, (**c**) PEF−5%−as, (**d**) PEF−10%−as, (**e**) PDoF−2.5%−as, (**f**) PDoF−5%−as, (**g**) PDoF−10%−as.

The microstructural features highlighted by LM microscopy are confirmed and expanded by the SEM analysis, whose results are shown in Figure 3a–g, representing the FESEM micrographs of the cross−sections of the as−spun fibers, and in Figure 4a–g, showing their lateral surface. First, from the micrographs of the cross−sections (Figure 3a–g), the difference in domain size between PEF and PDoF is here confirmed and quantified, as the PEF domain size is 10.0 ± 0.2 μm, while that of the PDoF domains is approx. one−tenth, being 0.7 ± 0.1 μm. Moreover, the cross−sections are characterized by a compact outer ring and a porous core. This morphology has probably been favored by the non−circular cross−sections of the fibers, which allows a quicker solvent release compared to a perfectly circular fiber, thus leading to a sheath–core structure and a higher inner porosity. All porosity is concentrated in the PLA matrix, while PEF and PDoF domains appear completely dense and homogeneous along the cross−section axis, which is probably due to their small dimensions. Finally, PLA and PEF domains show scarce compatibility and poor adhesion with the surrounding PLA matrix, being worse for PDoF than for PEF. This could partially explain the limited mechanical properties of these fibers (see Section 3.3). 

Figure 4a–g show the FESEM micrographs of the lateral surfaces of the as−spun fibers. The neat PLA fibers (Figure 4a) and the PLA/PDoF fibers (Figure 4e–g) present a relatively smooth lateral surface, whereas the PLA/PEF fibers (Figure 4b–d) present a remarkable surface roughness, which is in good agreement with what has been reported for the LM micrographs. Such a defective structure, similar to that observed by Gupta et al. [45] and Liu et al. [46] in polyacrylonitrile spun fibers, can be attributed to the different rates of solvent release upon the addition of PEF.

**Figure 5:**
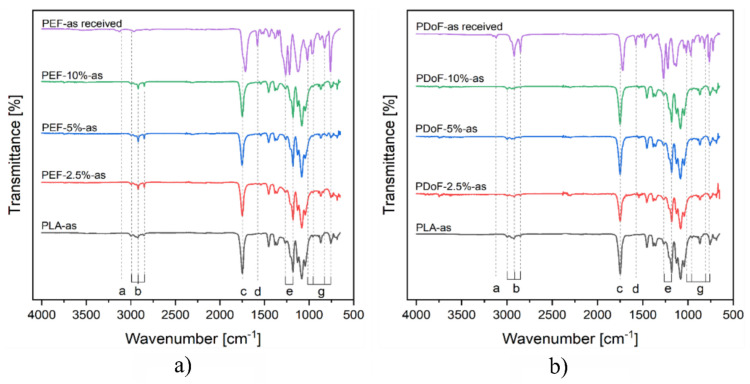
ATR−FTIR spectra of (**a**) PLA−as, PEF−2.5%−as, PEF−5%−as, PEF−10%−as, and PEF−as received samples, (**b**) PLA−as, PDoF−2.5%−as, PDoF−5%−as, PDoF−10%−as, and PDoF−as received samples.

The structural properties of the fibers were investigated with spectroscopic and diffraction techniques. Figure 5a,b show the ATR−FTIR spectra of the PLA, PEF−x−as and PDoF−x−as fiber blends (with x = 2.5%, 5%, or 10%), and of the bulk, PEF, and PDoF samples. Neat PLA fibers show a small signal corresponding to the CH_3_ out−of−phase and in−phase stretching at 2995 cm^−1^ and 2950 cm^−1^, respectively. The peaks at 1747 cm^−1^ and 1181 cm^−1^ are related to the C=O and C−O−C stretching frequencies, respectively. Moreover, CH_3_ out−of−phase and in−phase bending frequencies can be identified at 1453 cm^−1^ and 1380 cm^−1^, respectively. Moreover, two peaks can be detected in all the analyzed samples at 868 cm^−1^ and 755 cm^−1^, which are related to the crystalline and amorphous phases of PLA, respectively [22,47], which implies that the produced fibers are semi−crystalline. The regions of interest of the neat−PEF sample are two weak bands found at 3165 cm^−1^ and 3117 cm^−1^, which are related to the out−of−phase and in−phase C−H stretching of the furan ring, and a weak band at 2964 cm^−1^, which is related to the out−of−phase stretching of alkyl methylene groups, a strong absorption peak related to the C = O stretching centered at 1714 cm^−1^, a band at 1576 cm^−1^ linked to the C = C stretching vibration, the C−O in ester bond stretching vibration identified at 1260 cm^−1^, a furan ring breathing band centered at 1010 cm^−1^, and ring bending at 952 cm^−1^, 827 cm^−1^, and 751 cm^−1^ [48]. The regions of interest of PDoF are very similar to those related to PEF. The main assigned peaks are two weak bands found at 3160 and 3119 cm^−1^ related to the out−of−phase and in−phase C − H stretching of the furanic ring, and a weak band at 2918 and 2854 cm^−1^ related to the out−of−phase and in−phase stretching of alkyl methylene groups, a strong absorption peak related to the C = O stretching centered at 1714 cm^−1^, a band at 1570 cm^−1^ linked to the C = C stretching vibration, the C−O in ester bond stretching vibration identified at 1268 cm^−1^, a furan ring breathing band centered at 1018 cm^−1^, and ring bending at 966 cm^−1^, 824 cm^−1^, and 759 cm^−1^. 

The fiber blends do not show any peak related to the presence of PEF or PDoF, which is due to their limited weight fraction in the blends. Moreover, no red or blue shifts are highlighted when comparing the blends’ spectra with those of the neat−PEF and neat−PDoF, thus highlighting no relevant interactions between the polymer phases in the blends, which is similar to our previous findings [22,24,32]. 

Finally, X−ray diffraction (XRD) was performed to study the differences in microstructure and crystallinity between the as−spun and drawn fibers. In Figure 6a, the significant 2θ range of the powder diffraction pattern of PLA−as is reported, showing the two main (200) and (203) Bragg reflections located at 17° and 19°, respectively. In PLA/PAF fiber samples, no significant signals arise from the crystalline fractions of PEF and PDoF, which is likely due to their low volume fractions. For this reason, data analysis through the Rietveld method was carried out taking into account only the reference crystal structure of α−PLA reported in [49,50] and considering the preferred orientation and crystalline domain size refinement of the same; significant refined values are reported in Table 3. 

For preferred orientation, a satisfactory agreement with the data was obtained by means of one single Standard Function model with fiber symmetry; any further parameterization did not result in significant improvement in the fitting. Pole figure reconstruction from the Orientation Distribution Function demonstrates that the orientation of the crystallites happens preferentially with the (002) plane aligning along the fiber axis. Figure 6b shows the pole figures of the (200) plane for the PLA−as, PEF−10%−as, PDoF−10%−as, PLA−DR2, PEF−10%−DR2, and PDoF−10%−DR2, with the orientation density represented as a color map in the Multiple of Random Distributions (M.R.D.) scale. As expected, the drawing procedure generally increases the amount of the preferred orientation compared to the as−spun samples, with again the c−axis of the orthorhombic α−PLA cell preferentially aligning along the fiber axis. Interestingly, however, the addition of the PAFs significantly decreases the preferred orientation in both the as−spun and drawn samples; this could also contribute to the lower stiffness of the fiber blends compared to the neat PLA fibers.

To characterize the microstructure, only size−broadening effects were considered, due to the relatively low information content of the diffraction pattern in terms of well−defined Bragg reflections; the average volume−weighted domain size was refined in the limits of the isotropic approximation. As expected, the drawing procedure generally increases the dimensions of the crystallites in both neat PLA and PLA/PAF fibers; moreover, the latter exhibit slightly higher domain size values with respect to neat PLA to start with.

### 3.2. Thermal Properties

**Figure 7:**
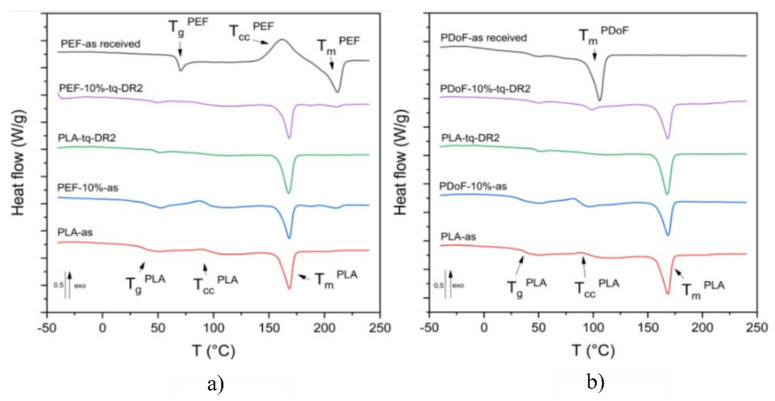
DSC first heating scan thermograms of: (**a**) PLA−as, PEF−10%−as, PEF−10%−DR2, and PEF−as received samples, and of (**b**) PLA−as, PDoF−10%−as, PDoF−10%−DR2, and PDoF−as received samples.

Figure 7a,b report the DSC thermograms of the prepared samples, while the main results are reported in Table 4. Figure 7a represents the DSC thermograms (first heating scan) of PLA−as, PEF−10%−as, PEF−10%−DR2, and PEF−as received. The neat−PEF sample presents a glass transition temperature at 68.8 °C, a cold crystallization phenomenon at 161.1 °C, and an endothermic melting peak at 211.6 °C, which is in good agreement with previous results from the literature [30]. The glass transition of the PLA−as sample was observed at 37.4 °C, which is a lower value below with respect to those found in the literature, which are equal to 55–60 °C [51]. Such a shift is probably linked to the plasticizing effect of moisture and the residual solvent. The small amplitude of the cold crystallization peak (approx. 85 °C) is probably due to the concurrent evaporation of the residual solvent. This could lead to a slight underestimation of the crystallinity degree of the sample, which has been found to be equal to 32%. The melting temperature for the PLA−as sample is 168 °C, which is consistent with literature values [51].

Regarding the PEF−10%−as and PEF−10%−DR2 samples, no major differences can be observed between the T_g_ of the blends and that of neat PLA. Additionally, three distinct melting phenomena were identified in both PEF−10%−as and PEF−10%−DR2 samples at 167.9 °C, 187.5 °C, and 210.9 °C corresponding to the α− and αʹ−crystalline phases of PLA and the crystalline portion of the PEF phase, respectively, thus confirming the immiscibility of the polymers in the crystalline state. Higher crystallinity values were found for the drawn samples compared to the as−spun ones, which is in good agreement with XRD results. Moreover, unlike what was observed with the XRD results, the addition of PEF leads to a lower crystallinity degree, which is likely due to the hindrance effect that the PEF domains induce on PLA chains.

Figure 7b shows the DSC first heating scan thermograms of PLA−as, PDoF−10%−as, PDoF−10%−DR2 fibers, and PDoF−as received. The PDoF−as received sample shows a broad melting event with a peak temperature of 105.5 °C and an associated crystallinity degree of 65 %. The same melting event can be observed in both PDoF−10%−as and PDoF−10%−DR2, where small melting peaks are detectable. Moreover, the values of T_g_, T_cc_, and T_m_ of PLA are not different from those of neat PLA fibers, which account for the blend immiscibility, which is in good agreement with the LM and FESEM micrographs and the ATR−FTIR spectra. As expected, the drawn fibers present higher crystallinity than the as−spun samples. In addition, for these blends, and unlike what has been observed with the XRD results, the addition of PDoF leads to lower crystallinity degrees compared to neat PLA, which is probably because of the constraint that the well−distributed domains impose on the PLA chains.

**Figure 8:**
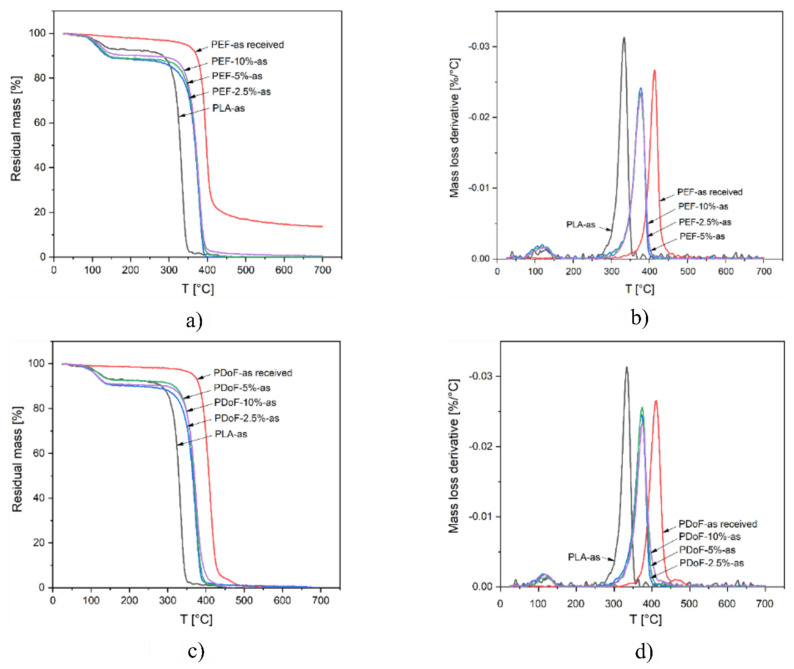
TGA thermograms: (**a**) residual mass and (**b**) mass loss derivative of the PLA−as, PEF−2.5%−as, PEF−5%−as, PEF−10%−as, and PEF−as received samples; (**c**) residual mass, and (**d**) mass loss derivative of PLA−as, PDoF−2.5%−as, PDoF−5%−as, PDoF−10%−as, and bulk PDoF−as received samples.

TGA tests were carried out to detect the effect on the thermal degradation resistance played by PAF addition and/or the drawing process. Figure 8a–d show the TGA thermograms and the derivative thermogravimetric (DTG) curves of PLA, PEF−x−as, and PDoF−x−as fiber blends (with x = 2.5%, 5%, or 10%), and the bulk, PEF, and PDoF samples as received, while the main results are reported in Table 5. The thermograms of PLA−as, PEF−x−as, and PDoF−x−as (Figure 8a,c) show a mass loss of 7–11 wt % starting at approx. 90 °C, which is associated with the evaporation of absorbed moisture and residual solvent. This phenomenon is not present in the as−received PEF and PDoF samples, which demonstrates their non−hygroscopic tendency. After this initial mass loss, all samples experience thermal degradation in a single step. For neat PLA, the maximum degradation speed is reached at approx. 333 °C (see T_D_ values in Table 5), while for both PAFs, it is at nearly 400 °C. As expected, the value of T_D_ for the blends is found at intermediate values, being 376–378 °C for the PEF−containing fibers and 372–374 °C for the PDoF−containing ones, but no specific trend can be observed as a function of the PAF fraction. A similar effect can be observed for the onset degradation temperature (T_onset_), which is increased from 318 °C of neat PLA to approx. 352 °C of the PLA/PAF blends. Interestingly, the height of the DTG peak (Figure 8b,d) of all blends is less intense than those of the corresponding parent polymers, which indicates a slower thermal degradation [52]. These results suggest that the addition of a small fraction of PEF or PDoF improves the thermal degradation resistance of PLA. 

### 3.3. Mechanical Properties

**Figure 9:**
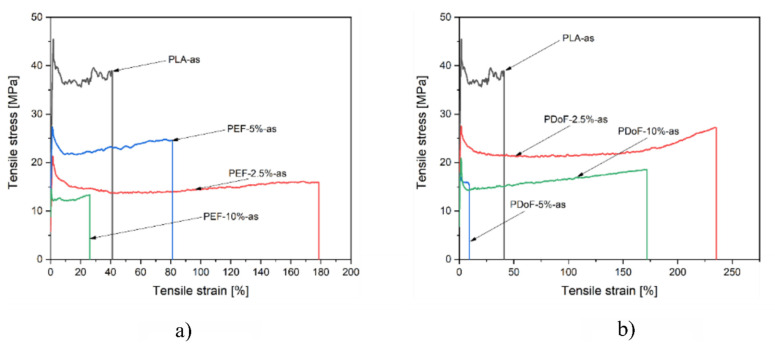
Representative stress–strain curves of the (**a**) PLA/PEF and (**b**) PLA/PDoF as−spun fiber blends.

The mechanical properties of the as−spun and drawn fibers were investigated through single−fiber tensile tests in quasi−static mode. Figure 9a,b report a representative stress–strain curve of the PLA−as, PEF−x−as, and PDoF−x−as (with x = 2.5%, 5%, or 10%.) fiber blends. PLA−as fibers are characterized by relatively high mechanical properties but a limited elongation at break, as expected. Interestingly, through the addition of a small quantity of both PEF and PDoF, the elongation at break of the produced fibers increases noticeably at the expense of the other mechanical properties. As expected, by increasing the amount of PEF and PDoF, a more brittle behavior characterizes the produced fibers. Given the fact that PLA and PEF or PDoF are not miscible, as shown in the microstructural and DSC analysis, the adhesion between the two polymeric phases may be limited, resulting thus in a decrease in the mechanical properties at elevated PAFs contents.

**Figure 10:**
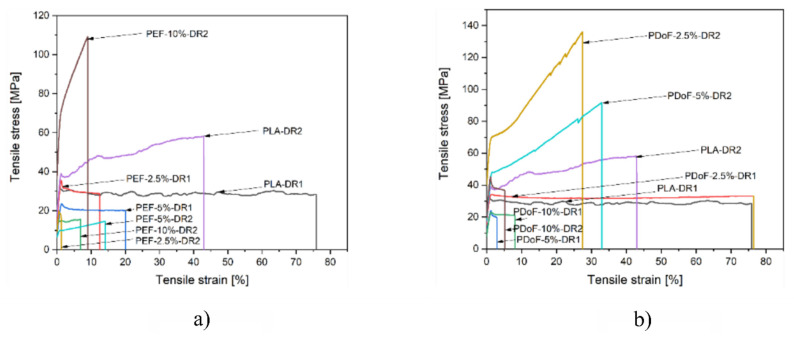
Representative stress–strain curves of the (**a**) PLA/PEF and (**b**) PLA/PDoF fiber blends drawn at DR = 1 and DR = 2.

Figure 10a,b report a representative stress–strain curve of the PLA−DR1 and DR2, PEF−x−DR1 and DR2, and PDoF−x−DR1 and DR2 (with x = 2.5%, 5%, or 10%.) fiber blends. The drawing procedure, as expected, improves the mechanical properties compared to the as−spun samples, and it can be appreciated in the compositions PEF−2.5%−DR1 and PDoF−2.5%−DR1. Samples with higher percentages of PEF and PDoF (5 wt % and 10 wt %) drawn with DR = 1 still show lower maximum stress and strain at break values than those of PLA−DR1. Interestingly, by increasing the DR from 1 to 2, the PLA/PEF fiber blends become quite brittle with limited elongation at break and maximum strength. On the other hand, PLA/PDoF fiber blends drawn with higher DR showed higher maximum strength at the expense of the elongation at break. The prepared fiber blends show average elastic modulus and maximum strength values comparable to those of PLA−DR1, which are equal to 2.25 GPa and 35.9 MPa, respectively. The lower values of the elastic modulus and maximum stress of the fiber containing PAFs may also be due to the porosity and lower adhesion between the constituents. The mechanical properties of the blends could be improved by further tuning the wet−spinning parameters and by means of a compatibilizer. The elongation at break values are lower than in the as−spun case: this could also be due to both the drawing procedure and the evaporation of the residual solvent during the drawing procedure; hence, no plasticizing effect occurred.

**Figure 11:**
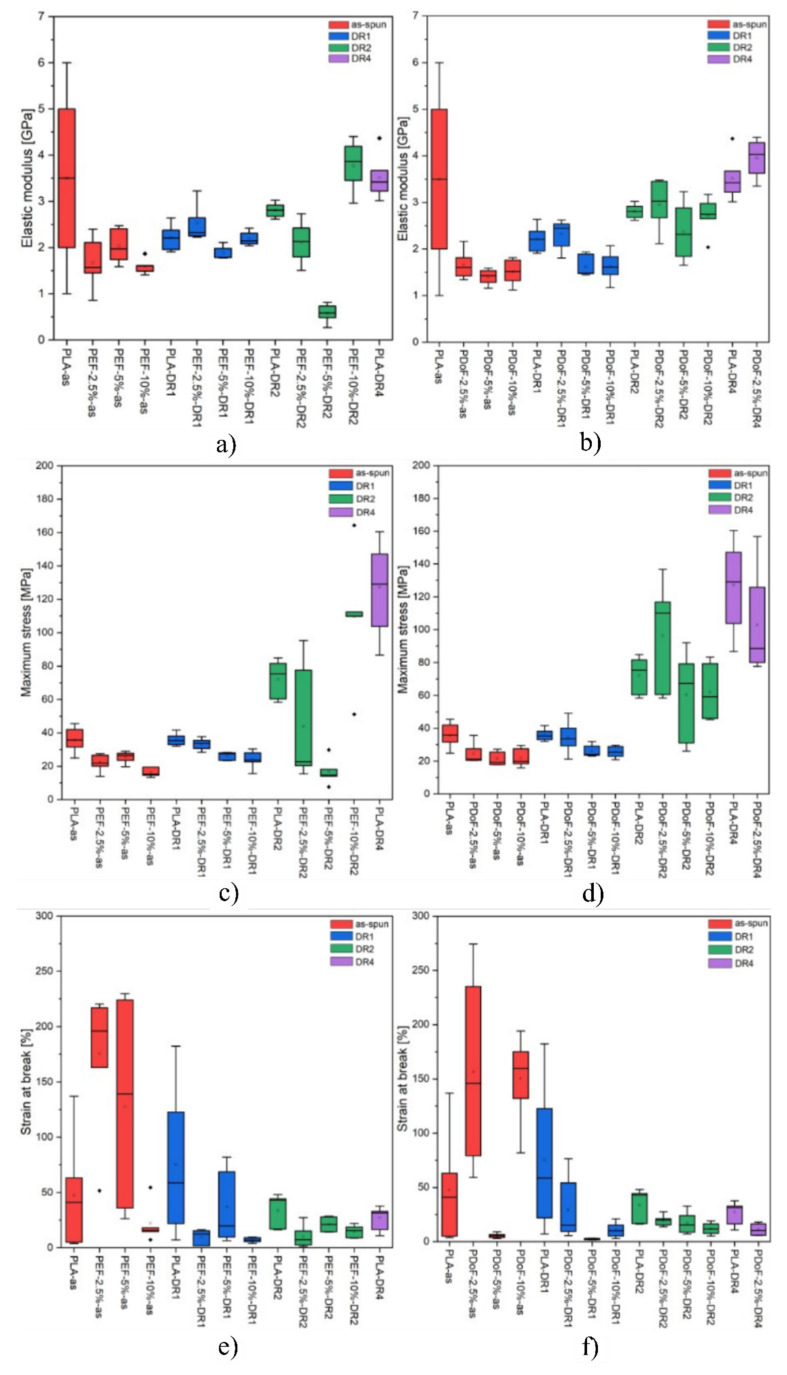
Boxplot of the samples grouped according to the composition and the drawing process: elastic modulus of (**a**) PLA/PEF and (**b**) PLA/PDoF fiber blends, maximum stress of (**c**) PLA/PEF and (**d**) PLA/PDoF fiber blends, strain at break and of (**e**) PLA/PEF and (**f**) PLA/PDoF fiber blends.

Figure 11a–f represent the boxplot plot of the elastic modulus and maximum stress of the samples grouped according to their composition and drawing procedure. Figure 11a,b shows the boxplots related to the elastic modulus of all the prepared fibers. As expected, the fibers show an increasing elastic modulus as the DR increases, which can be observed in the PDoF−2.5% samples as it presents an increase in the elastic modulus values at DR4 of approx. 41% with respect to the as spun case. Additionally, this sample also presents an improvement of 7% in the elastic modulus values compared to PLA drawn at the same draw ratio. The PAF domains in the as−spun samples possibly act as defects when included in larger percentages, also because of the low adhesion between PLA and the PAFs, as the elastic modulus values of the fiber blends were lower than that obtained for PLA. The increasing draw ratio elongated the PAF domains in the blends, leading to a domain size reduction of approx. 14%, thus possibly acting as a reinforcement of the PLA matrix. Moreover, the addition of furanoates does not significantly alter the elastic modulus values.

Figure 11c,d shows the boxplots related to the maximum stress of all the prepared fibers. The maximum tensile stress is 15 MPa lower (average values) for the as−spun fibers containing furanoates with respect to the PLA−as sample. By increasing the draw ratio, higher maximum stress values can be detected, as expected, although a higher scattering of the values is found. An improvement of up to 66% in the maximum stress values can be seen in the drawn fiber blends compared to the PLA−DR1 sample.

Figure 11e,f show the boxplots related to the strain at break of all the prepared fibers. High ultimate strain values are reached by the as−spun samples, with average deformations up to almost 200%, which is supposed to be due to the higher solvent content present in these specimens with larger diameters. As the draw ratio increases, the fiber stretching reduces the fiber diameters. Moreover, the drawing procedure was carried out at 80 °C, which is a sufficient temperature for the evaporation of the chloroform and/or HFIP, which could have still been present in the specimens, as seen in the TGA analysis. No consistent correlation d can be thus highlighted between the furanoate content in the blends and their deformation at break.

**Figure 12:**
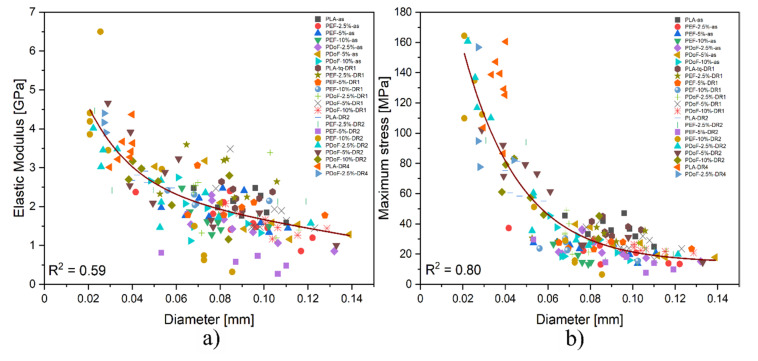
Trend of the (**a**) elastic modulus, and (**b**) maximum stress as a function of the diameter of all the prepared fibers.

It has to be considered that due to the experimental uncertainties connected to the preparation procedure, the final diameter of the fibers could strongly affect their mechanical properties. Therefore, a comprehensive analysis that correlates the stiffness and the strength of the obtained fibers with their diameter is required. Figure 12a,b show the trend of the elastic modulus and maximum stress as a function of the fiber diameters. Both the elastic modulus (E) and maximum stress (σ_max_) present an exponential dependence on the fiber diameter, with R^2^ values of 0.59 and 0.80, respectively, thus explaining the large scattering of the results obtained from the tensile tests. 

In addition to the effect played by the different DR applied, fibers presenting larger diameters show a plastic behavior because of the presence of residual solvent, whose presence was confirmed also by the TGA results. The samples with smaller dimensions contain less solvent, as they could be more easily removed. 

Table 6 summarizes the values of titer and tenacity of all the prepared fibers. Comparing the tenacity of the prepared PLA fibers with the literature values [53], those reported in this work are 10 times lower. Such a difference may be due to the high porosity of the fibers, the limited draw ratio applied, and the different spinning techniques adopted. Moreover, the addition of furanoates slightly lowered the tenacity values with respect to PLA fibers. This may be due to the non−circular geometry of the fibers and also to the low compatibility between the two, which may eventually have led to low adhesion. The tenacity of the drawn PLA fibers did not improve upon drawing, whilst a great improvement in the tenacity values can be observed for the fiber blends containing PAFs. The positive effect of the drawing procedure on the tenacity is probably due to the decrease in the overall diameters of the PAFs domains, together with the fiber’s diameters, and thus, they no longer act as defects.

**Figure 13:**
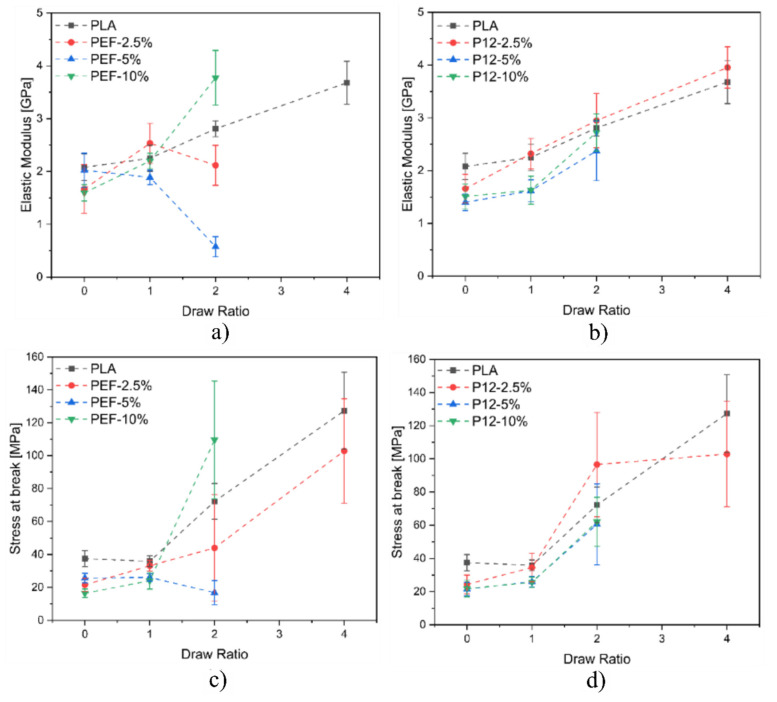
Plots of the elastic modulus as a function of the draw ratio (**a**) PLA/PEF and (**b**) PLA/PDoF fiber blends, and plot of stress at break as a function of the draw ratio (**c**) PLA/PEF and (**d**) PLA/PDoF fiber blends.

Figure 13a,b show the plots related to the elastic modulus of all the prepared fibers as a function of the DR, while Figure 13c,d show the plots related to the maximum stress of all the prepared fibers as a function of the DR. Concerning the blends containing PEF, it is possible to notice that the elastic modulus does not substantially increase by increasing the DR. On the other hand, the fiber blends PEF−10% reported a significant increase in the elastic modulus at DR = 2. As expected, PLA fibers report an increase in the elastic modulus as a function of the DR. Regarding PLA/PDoF fiber blends, it is possible to notice that the elastic modulus increases for all the compositions as a function of the DR. This could be explained by the different morphology of the fiber blends. PLA/PEF presents several domains of PEF distributed inside the PLA matrix, while PLA/PDoF reports small and homogenously distributed PDoF domains inside the PLA matrix. The improved domain distributions of PDoF fiber blends result in an improved distribution of the loads inside the fibers in comparison to the poorer domain distributions of PEF fiber blends. In conclusion, as expected, for both fiber blends, it is possible to notice that the stress at break increases by increasing the DR due to the improved alignment of the polymeric chains.

## 4. Conclusions

Bio−based fibers made of PLA and furan−based polyesters, namely PEF and PDoF, were successfully produced via an improved wet−spinning process, reaching a spinning draw ratio of 8. Additionally, the obtained fibers were drawn at three different draw ratios: 1, 2, and 4. A thorough characterization of the microstructural, chemical, thermal, and mechanical properties of the result in fibers was performed. 

LM micrographs highlighted the general elliptical geometry of the cross−section of the fibers and the immiscibility between the PLA and the PAFs phase. Such conclusions were confirmed by the FESEM micrographs, which also highlighted the presence of a compact outer ring and a porous fiber core, which was due to the evaporation of the solvent during the spinning process. A distinction between the PEF and PDoF domains was also observed, as the first were present as large and concentrated domains, whereas the second were small and well−distributed, with a decrease in their size as the distance from the fiber axis increased. Such a difference was most likely due to the difference in the solvent release rate between the PEF and PDoF. The lateral surface of the PLA and PLA/PDoF−x as−spun fibers appeared to be smooth, while that of the PLA/PEF as−spun fibers showed rough lateral surfaces, confirming thus the difference in the solvent release rates between PEF and PLA. 

The XRD analysis identified the presence of α−PLA as indicated by the presence of the two strongest Bragg reflections corresponding to (200) and (203) planes, which are located at 17° and 19°, respectively. Microstructural analysis performed by means of Rietveld modeling showed that the addition of the PAFs had positive effects on the degree of crystallinity, while on the other hand, it significantly decreased the preferred orientations in both the as−spun and drawn samples, as indicated by the reconstructed (200) PLA pole figures. Finally, the drawing procedure increased the dimensions of the crystallites and the preferred orientation compared to the as−spun samples. The ATR−FTIR spectra of the fiber blends only showed the presence of PLA due to the low PAF contents. No red or blue shifts were observed; hence, no new chemical bonds between the different polymers were formed, confirming thus that the blends were immiscible.

DSC analysis also proved the immiscibility of the blends, as no significant shifts in the T_g_ of PLA could be detected. The addition of the PAFs leads to a decrease in the crystallinity degree compared to neat PLA, although the PEF−containing samples showed higher values than those of the samples containing PDoF because of the smaller dimensions and uniform distribution of the PDoF domains. Additionally, the drawing procedure effectively increased the crystallinity. TGA tests revealed an improved thermal degradation stability upon the furanoate addition, in both the tested blends, without any clear correlation with the furanoate amount. 

Quasi−static tensile tests highlighted that the addition of poly(alkylene furanoate)s lowered the stress at break values of the as−spun fibers compared to the values of neat PLA fibers. The drawing process generally improved the mechanical properties of the fiber blends, with the elastic modulus and maximum stress values of the blends comparable or better than those of neat PLA. The elongation at break of the blends was higher than that of the as−spun PLA fibers, but it gradually decreased as the draw ratio increased. 

The present work optimized the production of fully bio−based wet−spun fibers. Compared to the previous work [24], higher draw ratios were reached, and the mechanical properties of the produced fibers were therefore improved. Moreover, the microstructure was improved as smoother lateral surfaces and less porosity were observed, and also the thermal stability and the crystallinity degree of these fibers was improved. In order to further increase the mechanical properties of these fibers, a proper compatibilizer may be added in the future to further improve the adhesion between the constituents, and further tuning of the spinning parameters could be performed in order to homogenize both fiber diameter and porosity degree.

## Figures and Tables

**Table 1:** List of the prepared samples with their nominal weight compositions.

	PAF Content	As Spun	Draw Ratio 1	Draw Ratio 2	Draw Ratio 4
	0 wt %	PLA−as	PLA−DR1	PLA−DR2	PLA−DR4
**PEF**	2.5 wt %	PEF−2.5%−as	PEF−2.5%−DR1	PEF−2.5%−DR2	PEF−2.5%DR4
	5 wt %	PEF−5%−as	PEF−5%−DR1	PEF−5%−DR2	PEF−5%−DR4
	10 wt %	PEF−10%−as	PEF−10%−DR1	PEF−10%−DR2	PEF−10%−DR4
**PDoF**	2.5 wt %	PDoF−2.5%−as	PDoF−2.5%−DR1	PDoF−2.5%−DR2	PDoF−2.5%−DR4
	5 wt %	PDoF−5%−as	PDoF−5%−DR1	PDoF−5%−DR2	PDoF−5%−DR4
	10 wt %	PDoF−10%−as	PDoF−10%−DR1	PDoF−10%−DR2	PDoF−10%−DR4

**Table 2:** Diameter and circularity of the as−spun fibers.

Sample	Fiber Diameter (μm)	Circularity
PLA−as	113 ± 23	0.80 ± 0.07
PEF−2.5%−as	66 ± 18	0.96 ± 0.04
PEF−5%−as	57 ± 16	0.95 ± 0.03
PEF−10%−as	60 ± 21	0.93 ± 0.06
PDoF−2.5%−as	57 ± 9	0.91 ± 0.05
PDoF−5%−as	92 ± 28	0.93 ± 0.04
PDoF−10%−as	65 ± 10	0.91 ± 0.04

**Table 3:** Semi−quantitative results of the XRD data modellization.

	PLA−as	PEF−10%−as	PDoF−10%−as	PLA−DR2	PEF−10%−DR2	PDoF−10%−DR2
**Average crystallite size (Å)**	311	331	322	332	390	368
**Preferred orientation (m.r.d)**	2.58	1.56	1.78	2.65	1.67	1.72

M.R.D. = multiples of a random distribution.

**Table 4:** Results of DSC tests of the PLA−as, PEF−10%−as, PEF−10%−DR2, and PEF−as received samples, and of PDoF−10%−as, PDoF−10%−DR2, and PDoF−as received samples.

Sample	T_g_^PLA^	T_g_^PAF^	T_cc_^PLA^	ΔH_cc_^PLA^	T_m_^PLA^	T_m_^PAF^	ΔH_m_^PLA1/2^	ΔH_m_^PAF^	χ_PLA_
	(°C)	(°C)	(°C)	(J/g)	(°C)	(°C)	(J/g)	(J/g)	(%)
First heating scan									
PLA−as	37.4	n.a.	89.9	7.1	168.0	n.a.	37.7	n.a.	32.6
PLA−DR2	47.3	n.a.	87.4		167.9	n.a.	34.9	n.a.	41.4
PEF−10%−as	39.8	−	87.4	14.0	167.9	210.9	32.4/0.8	3.3	21.9
PEF−10%−DR2	45.3	−	−	−	167.9	210.8	32.3/0.6	2.7	38.3
PEF−as received	n.a.	68.7	161.9 *	46.0 *	n.a.	211.6	n.a.	40.5	n.a.
PDoF−10%−as	31.7	−	82.0	14.6	168.8	−	34.3	−	23.3
PDoF−10%−DR2	44.4	−	−	−	167.9	−	35.6	−	25.0
PDoF−as received	n.a.	−	−	−	n.a.	105.5	n.a.	61.3	n.a.
Second heating scan									
PLA−as	57.3	n.a.	126.2	30.8	165.3	n.a.	31.8	n.a.	1.1
PLA−DR2	56.1	n.a.	121.8	32.5	165.2	n.a.	34.2	n.a.	1.8
PEF−10%−as	57.8	77.8	129.2	30.6	166.3	209.8	30.7	2.4	0.1
PEF−10%−DR2	57.8	−	129.3	27.9	165.7	209.5	29.8	3.3	2.2
PEF−as received	n.a.	75.3	170.7 *	25.7 *	n.a.	211.8	n.a.	25.7	n.a.
PDoF−10%−as	58.1	−	107.6	35.5	168.4	−	35.5	−	0.0
PDoF−10%−DR2	56.1	−	109.2	35.1	167.8	−	35.6	−	0.7
PDoF−as received	n.a.	−18.6	n.a.	n.a.	n.a.	104.0	n.a.	63.2	n.a.

* = values of PEF; − = not detectable; n.a. = not applicable.

**Table 5:** TGA results for the PLA−as, PEF−2.5%−as, PEF−5%−as, PEF−10%−as bulk PEF−as received samples, PDoF−2.5%−as, PDoF−5%−as, PDoF−10%−as and bulk PDoF−as received samples.

Sample	Δm_150 °C_ [%)	T_onset_ [°C)	T_D_ [°C)	m_final_ [%)
PLA−as	7.0	317.9	333.5	0.00
PEF−2.5%−as	11.6	354.0	377.5	0.00
PEF−5%−as	11.1	354.8	376.2	0.00
PEF−10%−as	9.8	352.7	378.2	0.55
PEF−as received	−	383.9	396.8	13.65
PDoF−2.5%−as	10.2	351.1	372.3	0.05
PDoF−5%−as	7.3	354.9	373.8	0.00
PDoF−10%−as	9.3	352.7	374.0	0.00
PDoF−as received	−	392.3	411.0	0.10

**Table 6:** Titer and tenacity values of the fibers blends (as−spun and drawn at DR = 2).

Sample	Titer (g/km) = (tex)	Tenacity (cN/tex)
PLA−as	6.00	3.98 ± 0.01
PEF 2.5%−as	5.06	2.54 ± 0.01
PEF 5%−as	4.94	2.57 ± 0.01
PEF 10%−as	4.59	1.76 ± 0.01
PDoF 2.5%−as	5.29	2.61 ± 0.01
PDoF 5%−as	5.77	3.47 ± 0.01
PDoF 10%−as	6.24	1.95 ± 0.01
PLA−DR2	2.71	4.02 ± 0.01
PEF 10%−DR2	1.54	4.15 ± 0.01
PDoF 10%−DR2	3.00	4.41 ± 0.01

## Data Availability

Data supporting the findings of this study are available on request by the corresponding author.
